# CXCL17 Expression Predicts Poor Prognosis and Correlates with Adverse Immune Infiltration in Hepatocellular Carcinoma

**DOI:** 10.1371/journal.pone.0110064

**Published:** 2014-10-10

**Authors:** Li Li, Jing Yan, Jing Xu, Chao-Qun Liu, Zuo-Jun Zhen, Huan-Wei Chen, Yong Ji, Zhi-Peng Wu, Jian-Yuan Hu, Limin Zheng, Wan Yee Lau

**Affiliations:** 1 Department of Hepatic and Pancreatic Surgery, The First People’s Hospital of Foshan, Foshan, Guangdong, P. R. China; 2 State Key Laboratory of Oncology in South China, Sun Yat-sen University Cancer Center, Guangzhou, P. R. China; 3 Faculty of Medicine, The Chinese University of Hong Kong, Shatin, New Territories, Hong Kong SAR, P. R. China; University of Leicester, United Kingdom

## Abstract

CXC ligand 17 (CXCL17) is a novel CXC chemokine whose clinical significance remains largely unknown. In the present study, we characterized the prognostic value of CXCL17 in patients with hepatocellular carcinoma (HCC) and evaluated the association of CXCL17 with immune infiltration. We examined CXCL17 expression in 227 HCC tissue specimens by immunohistochemical staining, and correlated CXCL17 expression patterns with clinicopathological features, prognosis, and immune infiltrate density (CD4 T cells, CD8 T cells, B cells, natural killer cells, neutrophils, macrophages). Kaplan-Meier survival analysis showed that both increased intratumoral CXCL17 (*P* = 0.015 for overall survival [OS], *P* = 0.003 for recurrence-free survival [RFS]) and peritumoral CXCL17 (*P* = 0.002 for OS, *P*<0.001 for RFS) were associated with shorter OS and RFS. Patients in the CXCL17^low^ group had significantly lower 5-year recurrence rate compared with patients in the CXCL17^high^ group (peritumoral: 53.1% vs. 77.7%, *P*<0.001, intratumoral: 58.6% vs. 73.0%, *P* = 0.001, respectively). Multivariate Cox proportional hazards analysis identified peritumoral CXCL17 as an independent prognostic factor for both OS (hazard ratio [HR] = 2.066, 95% confidence interval [CI] = 1.296–3.292, *P* = 0.002) and RFS (HR = 1.844, 95% CI = 1.218–2.793, *P* = 0.004). Moreover, CXCL17 expression was associated with more CD68 and less CD4 cell infiltration (both *P*<0.05). The combination of CXCL17 density and immune infiltration could be used to further classify patients into subsets with different prognosis for RFS. Our results provide the first evidence that tumor-infiltrating CXCL17^+^ cell density is an independent prognostic factor that predicts both OS and RFS in HCC. CXCL17 production correlated with adverse immune infiltration and might be an important target for anti-HCC therapies.

## Introduction

Hepatocellular carcinoma (HCC) is among the most common causes of cancer mortality worldwide and its incidence is increasing [1]–[3]. Although surgical resection is one of the first priorities for HCC treatment, the frequent postsurgical recurrence is a major obstacle to cure. Besides, the general response of HCC to chemotherapy remains far from satisfactory [4]. Thus, discovering biomarkers that identify patients at high risk for recurrence and combining other postsurgical adjuvant therapies to obtain better outcomes is of great importance [5].

Tumor progression is now recognized as the product of evolving crosstalk among different cell types within the tumor microenvironment. Extensive immune cell infiltration is usually present in HCC due to chronic viral infection [6]–[8]. Chemokines are key mediators for recruiting various immune cells to the tumor microenvironment. The infiltrating cells provide a secondary source of chemokines that further regulate the immune status of the tumor [9]–[12]. In addition to promoting leukocyte trafficking to tumors, recent studies indicate that chemokines also play important roles in many aspects, including influencing the tumor immune response, regulating angiogenesis, promoting tumor growth, and mediating tumor metastasis [13], [14]. The CXC ligand 5 (CXCL5) and CXC receptor 6 (CXCR6) expression has been shown to significantly influences HCC progression, in part by regulating tumor growth and invasion, and via immune cell infiltration [15], [16].

CXCL17 is a novel, 119-amino acid CXC chemokine; its receptor is currently unknown [17], [18]. As the last chemokine ligand to be discovered, the clinical significance and regulatory activity of CXCL17 remain largely unknown. CXCL17 is expressed constitutively in the lung, trachea, stomach and colon [17], [18]. It is a chemoattractant of monocytes, macrophages, dendritic cells (DC), and immature myeloid-derived cells [18]. Several studies have proposed that CXCL17 might act as a chemokine that accelerates tumor progression. CXCL17 is significantly upregulated in breast carcinoma and colon tumors and is associated with carcinogenesis, tumor proliferation, and angiogenesis [17], [19]. Interestingly, another study suggested that CXCL17 might be involved in anti-tumor immune response during pancreatic carcinogenesis. In premalignant intraductal papillary mucinous neoplasm, CXCL17 induces DC accumulation at the tumor site, which promotes tumor cell susceptibility to cytotoxic T cell-mediated cytolysis [20]. Although ectopic CXCL17 expression promotes hepatoma HepG2 cell proliferation, the nature and role of CXCL17 in human HCC has not been elucidated [21].

In the present study, we investigated the prognostic significance of CXCL17 in HCC. We then explored the possible regulatory activity of CXCL17 by correlating CXCL17 expression with immune cell infiltration. We also tried to find out whether tumor-infiltrating CXCL17^+^ cells were an independent prognostic factor of survival and whether they were correlated with immune infiltration in HCC.

## Materials and Methods

### Patients and Specimens

We used archived formalin-fixed, paraffin-embedded tissues from 8 patients with chronic hepatitis and 227 patients who had undergone curative resection for HCC between 2007 and 2010 at the Sun Yat-sen University Cancer Center. The diagnosis of HCC was confirmed histopathologically in each patient. No patient received anti-cancer therapies or had distant metastasis prior to surgery. Curative resection for HCC was defined as complete resection of all tumor nodules, with a resection margin of at least 1 cm, and leaving the cut surface being free of tumor based on histological examination. Intraoperative ultrasound and postsurgical contrast-enhanced computed tomography (CT) were routinely used to ensure complete removal of HCC [7], [22]. Tumor stages were determined according to the tumor-nodes-metastasis (TNM) classification of the International Union Against Cancer (Edition 6) and the Barcelona Clinic Liver Cancer (BCLC) staging classification [23]. Tumor differentiation was graded according to the Edmondson grading system. The clinicopathological characteristics of the patients were summarized in Table 1.

**Table 1:** Clinical characteristics of 227 patients with HCC.

Variables	Group 1 (CXCL17)	Group 2 (TMA)
Cases (n)	227	101
Age, years (median, range)	51, 13–79	52, 23–73
Gender (male/female)	197/30	90/11
HBsAg (negative/positive)	18/209	1/100
Cirrhosis (absent/present)	85/142	38/63
ALT (U/liter, ≤42/>42)	138/89	60/41
AST (U/liter, ≤42/>42)	131/96	60/41
AFP (ng/ml, ≤25/>25)	78/149	29/72
Tumor size (cm, ≤5/>5)	96/131	37/64
Tumor differentiation (I–II/III–IV)	129/98	44/57
Vascular invasion (absent/present)	186/41	83/18
Tumor multiplicity (solitary/multiple)	170/57	71/30
Capsulation (absent/present)	52/175	23/78
TNM stage (I–II/III–IV)	122/105	51/50
BCLC stage (0–A vs. B–C)	136/91	57/44

Abbreviations: AFP: alpha-fetoprotein; ALT: alanine aminotransferase; AST: aspartate aminotransferase; BCLC: Barcelona Clinic Liver Cancer; HBsAg: hepatitis B surface antigen; TMA: Tissue microarray; TNM: tumor-nodes-metastasis.

### Follow-up and Postoperative Treatment

All patients were followed-up after surgery, undergoing regular surveillance for recurrence with chest radiography, serum alpha-fetoprotein (AFP) level, and abdominal ultrasonography at 2–4-month intervals [7], [22], [24], [25]. If tumor recurrence or metastasis was suspected, further examinations, including CT, magnetic resources imaging (MRI) and hepatic angiography, were performed. Biopsies were obtained when necessary. Patients with confirmed recurrence received further treatment, such as a second surgical resection, radiofrequency ablation, transcatheter arterial chemoembolization, or percutaneous ethanol injection. The median follow-up was 36 months (range, 2–83 months). Of the 227 patients, 97 patients (42.7%) died, 143 patients (63.0%) were diagnosed with tumor recurrence, and 84 patients (37.0%) remained alive without recurrence on follow-up. Data were censored at the last follow-up for patients without recurrence or death. Overall survival (OS) was defined as the interval between the dates of surgery and the date of death or the last follow-up. Recurrence-free survival (RFS) was defined as the interval between the dates of surgery and recurrence or the last follow-up if no recurrence was observed.

### Ethics Statement

Written informed consents were obtained from all patients. All samples were coded anonymously, in strict accordance with local ethical guidelines and as stipulated by the Declaration of Helsinki. Before the study, the protocol was approved by the Research Ethics Committee of Sun Yat-sen University Cancer Center.

### Tissue Microarray, Immunohistochemistry and Immunocytochemistry

Tissue microarray (TMA) was constructed as described previously [22], [24]. Briefly, representative areas away from necrotic and hemorrhagic materials were premarked in the paraffin-embedded blocks by hematoxylin-eosin staining. Duplicates of 1-mm diameter cylinders from the center of the tumor and nontumor liver tissues were obtained from each case to ensure reproducibility and homogeneity. Immunohistochemistry (IHC) for CXCL17 was carried out on paraffin-embedded sections from the 227 patients to better define the anatomical localization of CXCL17 signals. IHC for CD4 (CD4 T cells), CD8 (CD8 T cells), CD20 (B cells), CD57 (natural killer cells), CD15 (neutrophils), and CD68 (macrophages) was performed on TMA sections from 101 specimens randomly selected from the 227 specimens.

IHC and immunocytochemistry (ICC) was carried out using a 2-step protocol (DakoCytomation, Glostrup, Denmark). For IHC, after dewaxing, hydration and endogenous peroxidase blocking was carried out (0.3% H_2_O_2_ for 10 min). Antigen retrieval was performed by steaming the sections in 10 mM citrate buffer (pH 6.0) for 10 min. Sections were incubated overnight at 4°C with mouse anti-CXCL17 (Monoclonal Mouse IgG2B; clone 422208; R&D Systems, Minneapolis, MN) anti-CD4, anti-CD8, anti-CD57 (Neomarkers, Fremont, CA), anti-CD20, anti-CD15 (Beijing Zhongshan Golden Bridge Biotechnology, Beijing, China), and anti-CD68 (DakoCytomation) antibodies. Horseradish peroxidase-conjugated anti-mouse and anti-rabbit Dako EnVision systems (DakoCytomation) were used as secondary detection reagents and developed with 3,3′-diaminobenzidine (DAB). All sections were counterstained with Mayer’s hematoxylin and mounted in non-aqueous mounting medium. Appropriate negative controls were used, in which the primary antibodies were replaced by the same concentration of irrelevant, isotype-matched antibodies. For ICC, leukocytes were isolated from peripheral blood of healthy donors. Density-gradient separation on polymorphprep was performed. The pale-red granulocyte layer was washed and the contaminated erythrocytes were lysed by a brief hypotonic lysis. The cells were then cytospun and cultured in medium alone or with 20% culture supernatants from hepatoma cell-line cells (HepG2 and Huh7) or LPS (50 ng/mL) respectively for 12 h. CXCL17 was stained as IHC staining as described above.

### Immunofluorescent Staining and Quantification

Paraffin-embedded HCC sections were incubated overnight at 4°C with primary mouse anti-CXCL17 and rabbit anti-myeloperoxidase (MPO; DakoCytomation), followed by Alexa Fluor 488 donkey anti-mouse and Alexa Fluor 555 donkey anti-rabbit antibodies, respectively (Invitrogen, Carlsbad, CA). Nuclei were stained with 4′,6-diamidino-2-phenylindole (DAPI). Slides were mounted with anti-fade mounting medium, dried for 24 h at room temperature, and then stored at −20°C for future use. Images were captured and analyzed using an Olympus FV1000 confocal laser scanning microscope (Center Valley, PA). To quantify the proportion of CXCL17^+^ cells, numbers of single-positive or double-positive cells in each of five representative fields at ×400 magnification were counted manually by two independent, blinded observers.

### Automated Image Acquisition and Quantification of IHC Staining

To detect and evaluate the CXCL17 signal in HCC tumors without bias, we established an automated and standardized quantification method using the Vectra-Inform image analysis system (Perkin-Elmer Applied Biosystems, Foster City, CA) [26]–[28]. Stained sections were captured with the Nuance VIS-FL Multispectral Imaging System and analyzed with InForm 2.0.1 image analysis software (Perkin-Elmer Applied Biosystems). Images were acquired at 440–700 nm at 20-nm intervals. The spectrum for each chromogen was determined on single-stained control slides and was analyzed by multispectral imaging analysis. A spectral unmixing algorithm separated the grayscale images representing each spectral component quantitatively (Fig. S1A–D). The InForm software enabled tissue compartment (tumor tissue, peritumoral stroma tissue, blank) and cell compartment (cytoplasm, nucleus) segmentation (Fig. S1E–H). The DAB object density counts per megapixel for each tissue category were used for further analysis. Examination of immune infiltrate densities (CD4, CD8, CD20, CD57, CD15, CD68) on TMA were also performed using a method similar to that established for quantifying CXCL17. The total number of CD4, CD8, CD20, CD57, CD15, and CD68 positive cells was deemed identified as the total immune cell infiltrations in the tissue. The percentage of each immune cell subset was calculated by dividing the absolute number of each subset by the total numbers of all these cells.

### Statistical Analysis

Subgroups of each immunostaining parameter were divided by their median values. Correlations between immunostaining parameters and clinicopathological features were analyzed by χ^2^ tests or Fisher’s exact test where appropriate. The association between the numbers of tumor-infiltrating immune cells and CXCL17 was calculated with the Pearson test. Survival analysis was carried out with the Kaplan-Meier method and was compared using the log-rank test. A multivariate Cox proportional hazards regression model was applied to estimate the adjusted hazard ratio (HR) and 95% confidence interval (CI) and to identify independent prognostic factors. For each analysis, 2-sided *P*<0.05 was considered statistically significant. All statistical analyses were performed with SPSS 17.0.

## Results

### CXCL17 Expression in HCC Tissue

**Figure 1:**
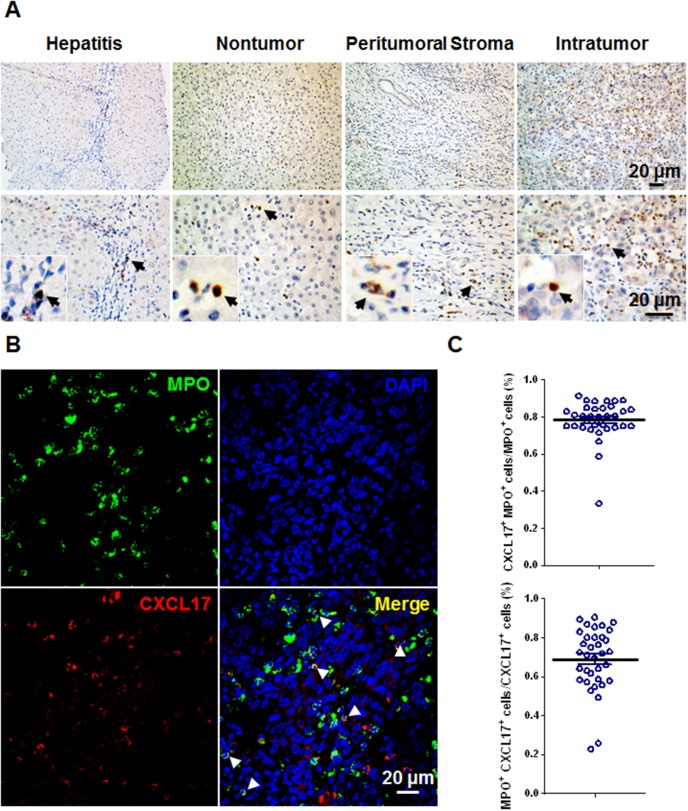
CXCL17 expression *in situ* in HCC tumors. (A) Representative sites depicting CXCL17-producing cells stained brown in human chronic hepatitis liver, nontumor, peritumoral stroma, and intratumoral regions in HCC. Representative sites with low (upper panels) and high (lower panels) magnification were shown. Black arrows indicated CXCL17^+^ cells. (B) Multiple staining of MPO (green), CXCL17 (red), and DAPI (blue, nuclei) in paraffin-embedded sections analyzed by confocal microscopy. The coexistence of MPO and CXCL17 confirmed that a proportion of MPO^+^ neutrophils expressed CXCL17. White arrows indicated representative neutrophils expressed CXCL17. (C) Proportions of CXCL17^+^MPO^+^ cells in CXCL17^+^ cells or MPO^+^ cells of HCC tissue. Results are expressed as mean ± SEM (bars).

We performed IHC staining of CXCL17 in paraffin-embedded sections to detect CXCL17 in HCC tumors. CXCL17 signals were mainly located in the cytoplasm of stromal cells, but were also observed occasionally in the cytoplasm or nuclei of tumor cells (Fig. 1A). There were CXCL17-producing cells in the nontumor, peritumoral stroma, and intratumoral regions with mean (± SEM) density of 100.0±12.06, 100.5±8.65, 99.3±12.07, respectively (Fig. 1A). The CXCL17 expression was also been examined on human hepatitis tissues (n = 8) with mean (± SEM) density of 29.05±11.13 (Fig. 1A). To determine the source of CXCL17 in HCC tissue, we performed double staining for CXCL17 and various immune cell markers. Confocal microscopic analysis showed that CXCL17 was mainly produced by MPO-positive neutrophils (68.8%±2.7%), and most of MPO-positive neutrophils produced CXCL17 (78.3%±1.8%) (Fig. 1C). Stromal cells with mononuclear morphology and tumor cells also produced CXCL17 (Fig. 1B).

### Increased CXCL17 Predicted Poor Survival

To determine whether CXCL17 can be a prognostic marker for HCC, we plotted Kaplan-Meier survival curves to investigate the correlation between CXCL17 expression and survival. Follow-up was completed on January 8, 2014, with a median follow-up duration of 36 months (range, 2–83 months). The 1-, 3-, 5-year OS and RFS rates were 82.7%, 61.8%, 51.2% and 59.9%, 39.2%, 34.4%, respectively. The CXCL17^high^ and CXCL17^low^ subgroups were divided by the median CXCL17 object density per megapixel (median intratumoral, peritumoral, nontumoral CXCL17 = 20.7, 46.4, 30.1, respectively).

**Figure 2:**
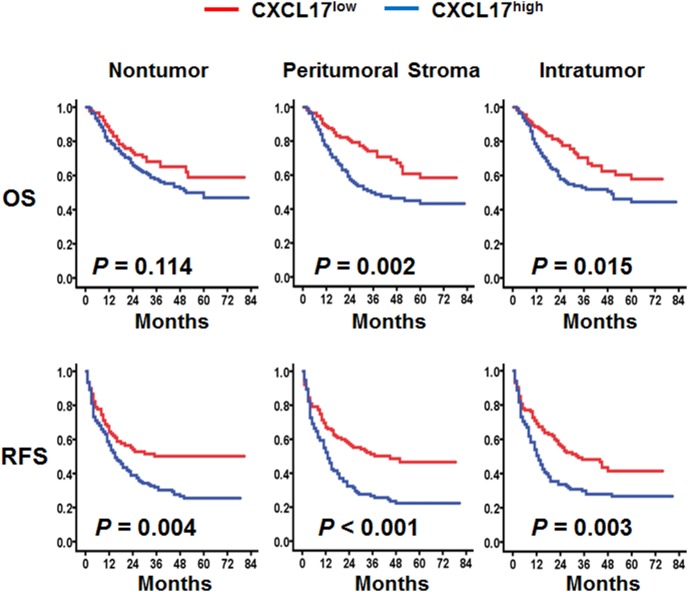
Accumulation of CXCL17-producing cells predicted poor survival in HCC. OS (top) and RFS (bottom) were estimated by the Kaplan-Meier method and compared using the log-rank test (n = 227).

Univariate analysis revealed that both increased intratumoral CXCL17 (*P* = 0.017 for OS, *P* = 0.005 for RFS, Table 2) and higher peritumoral CXCL17 (*P* = 0.002 for OS, *P*<0.001 for RFS, Table 2) were associated with shorter OS (intratumoral: 50 months vs. >72 months; peritumoral: 35 months vs. >72 months, respectively) and RFS (intratumoral: 13 months vs. 33 months; peritumoral: 13 months vs. 45 months, respectively). Patients in the CXCL17^low^ group had a significantly lower 5-year recurrence rate compared with patients in the CXCL17^high^ group (peritumoral: 53.1% vs. 77.7%, *P*<0.001, intratumoral: 58.6% vs. 73.0%, *P* = 0.003, log-rank test, Fig. 2). When stratified by tumor multiplicity, the peritumoral CXCL17 density remained a good predictor of OS (all *P*<0.05, Fig. S5). Moreover, regardless of tumor differentiation grade, tumor size, TNM stage, and aspartate transaminase (AST) and AFP levels, peritumoral CXCL17 was a good predictor of recurrence (all *P*<0.05, Fig. S5).

**Table 2:** Univariate and multivariate analyses of factors associated with survival and recurrence.

	OS				RFS			
	Univariate	Multivariate			Univariate	Multivariate		
Variables	*P*	HR	95% CI	*P*	*P*	HR	95% CI	*P*
Age (>51 vs. ≤51 years)	0.413			NA	0.145			NA
Gender (male vs. female)	0.755			NA	0.261			NA
HBsAg (positive vs. negative)	0.533			NA	0.079			NA
Cirrhosis (present vs. absent)	0.321			NA	0.310			NA
ALT (>42 vs. ≤42 U/L)	0.250			NA	0.620			NA
AST (>42 vs. ≤42 U/L)	0.003	1.468	0.960–2.246	0.077	0.010	1.348	0.948–1.917	0.097
AFP (>25 vs. ≤25 ng/ml)	0.001	2.181	1.331–3.574	0.002	0.004	1.738	1.190–2.540	0.004
Tumor size (>5 vs. ≤5 cm)	0.005	1.367	0.841–2.224	0.208	0.008	1.215	0.818–1.803	0.334
Tumor differentiation (III–IV vs. I–II)	0.154			NA	0.354			NA
Vascular invasion (present vs. absent)	0.001	1.367	0.706–2.645	0.354	0.025	1.110	0.626–1.966	0.721
Tumor multiplicity (multiple vs. solitary)	≤0.001	1.269	0.647–2.490	0.488	≤0.001	1.515	0.859–2.670	0.151
Capsulation (present vs. absent)	0.107			NA	0.073			NA
TNM stage (III–IV vs. I–II)	≤0.001	1.031	0.593–1.794	0.914	0.001	1.025	0.646–1.627	0.915
BCLC stage (B–C vs. 0–A)	≤0.001	1.656	0.737–3.718	0.222	≤0.001	1.352	0.699–2.614	0.370
Nontumoral CXCL17 (high vs. low)	0.118			NA	0.003	1.289	0.868–1.916	0.208
Peritumoral CXCL17 (high vs. low)	0.002	2.066	1.296–3.292	0.002	≤0.001	1.844	1.218–2.793	0.004
Intratumoral CXCL17 (high vs. low)	0.017	1.344	0.845–2.137	0.212	0.005	1.223	0.836–1.788	0.299

Note: Cox proportional hazards regression model was used. Variables used in multivariate analysis were adopted by univariate analysis. Underlined terms indicate statistical significance. Abbreviations: AFP: alpha-fetoprotein; ALT: alanine aminotransferase; AST: aspartate aminotransferase; BCLC: Barcelona Clinic Liver Cancer; CI: confidence interval; HBsAg: hepatitis B surface antigen; HR: hazard ratio; NA: not adopted; TNM: tumor-nodes-metastasis.

We next assessed whether CXCL17 could serve as an independent predictor of survival by performing multivariate Cox proportional hazards analysis. Clinicopathological variables demonstrated to be significant in the univariate analysis were used as covariates, and peritumoral CXCL17 was found to be an independent prognostic factor for both OS and RFS (Table 2). Peritumoral CXCL17 was associated with elevated risk of death (HR = 2.066, 95% CI = 1.296–3.292, *P* = 0.002) and recurrence (HR = 1.844, 95% CI = 1.218–2.793, *P* = 0.004). Other than CXCL17, the multivariate Cox proportional hazards analysis demonstrated that serum AFP level was an independent predictor of both OS and RFS (*P* = 0.002, *P* = 0.002, respectively).

To evaluate the correlation between CXCL17 expression and tumor development, we also analyzed the association between CXCL17 expression and the clinicopathological parameters. Table 3 shows that increased peritumoral CXCL17 significantly correlated with presence of liver cirrhosis (*P* = 0.045) and less advanced tumor differentiation (*P* = 0.004).

**Table 3:** Association of CXCL17 with clinicopathological characteristics.

		Nontumoral CXCL17			Peritumoral CXCL17			Intratumoral CXCL17		
Variable		Low	High	*P*	Low	High	*P*	Low	High	*P*
Age (year)	≤51	58	58	0.946	62	54	0.320	60	56	0.549
	>51	56	55		52	59		53	58	
Gender	Male	99	98	0.979	100	97	0.676	98	99	0.979
	Female	15	15		14	16		15	15	
HBsAg	Negtive	8	10	0.610	9	9	0.984	9	9	0.984
	Positive	106	103		105	104		104	105	
Cirrhosis	Absent	47	38	0.237	50	35	0.045	49	36	0.067
	Present	67	75		64	78		64	78	
ALT (U/liter)	≤42	67	71	0.531	68	70	0.723	70	68	0.723
	>42	47	42		46	43		43	46	
AST (U/liter)	≤42	62	69	0.309	60	71	0.120	64	67	0.745
	>42	52	44		54	42		49	47	
AFP (ng/ml)	≤25	38	40	0.743	35	43	0.244	33	45	0.103
	>25	76	73		79	70		80	69	
Tumor size (cm)	≤5	51	45	0.454	48	48	0.955	49	47	0.745
	>5	63	68		66	65		64	67	
Tumor differentiation	I + II	58	71	0.069	54	75	0.004	63	66	0.745
	III + IV	56	42		60	38		50	48	
Vascular invasion	Absent	93	93	0.888	95	91	0.583	95	91	0.406
	Present	21	20		19	22		18	23	
Tumor multiplicity	Solitary	83	87	0.467	83	87	0.467	84	86	0.848
	Multiple	31	26		31	26		29	28	
Capsulation	Absent	23	29	0.325	21	31	0.106	23	29	0.362
	Present	91	84		93	82		90	85	
TNM stage	I + II	67	55	0.127	68	54	0.073	66	56	0.161
	III + IV	47	58		46	59		47	58	
BCLC stage	0–A	68	68	0.935	69	67	0.850	69	67	0.725
	B–C	46	45		45	46		44	47	

Note: Underlined terms indicate statistical significance. Abbreviations: AFP: alpha-fetoprotein; ALT: alanine aminotransferase; AST: aspartate aminotransferase; BCLC: Barcelona Clinic Liver Cancer; CI: confidence interval; HBsAg: hepatitis B surface antigen; TNM: tumor-nodes-metastasis.

### Association between CXCL17 Expression and Local Immune Cell Infiltration

**Figure 3:**
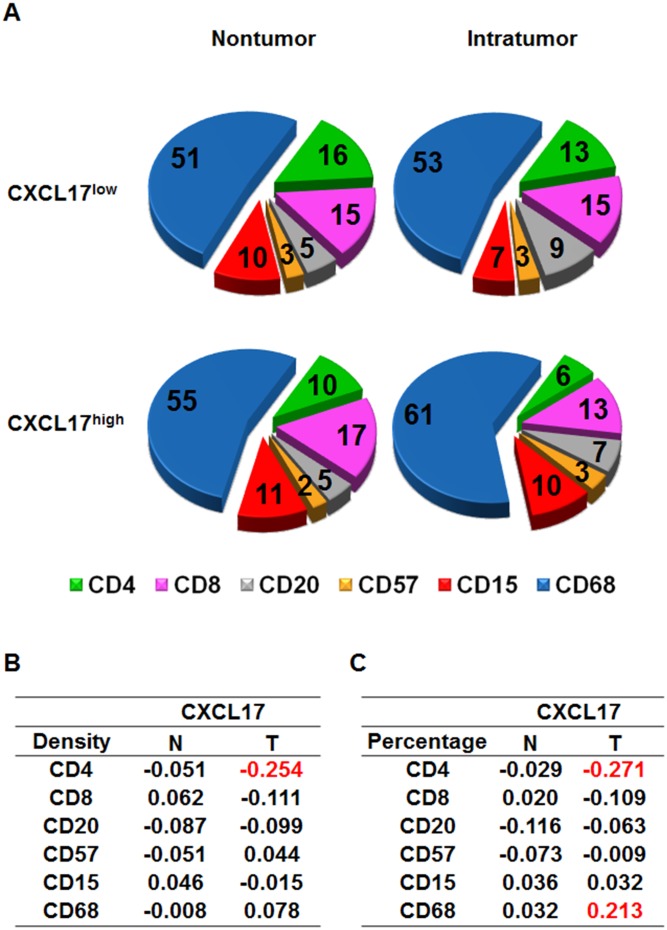
Composition of immune infiltrates according to CXCL17 expression. (A) Pie charts summarized the percentages of nontumor-infiltrating (N, left) and tumor-infiltrating (T, right) CD4, CD8, CD20, CD57, CD15, and CD68 cells in CXCL17^low^ and CXCL17^high^ groups. (B, C) Correlation coefficients between the density or percentage of CXCL17 and the density of each immune cell subset were shown.

Chemokines are best known for their ability to recruit cells by forming chemokine gradients. To determine the potential role of CXCL17 in immune cell regulation in HCC, we performed comprehensive analyses of immune cell infiltration and examined the correlation between CXCL17 expression and CD4^+^, CD8^+^, CD20^+^, CD57^+^, CD15^+^, and CD68^+^ cell density in HCC tissues using the Pearson correlation test. There was a negative correlation between the intracellular number of CD4 cells and CXCL17 expression (correlation coefficient, R = −0.254, *P*<0.05, Fig. 3B). Using the percentage of each immune cell subset in the total immune cell infiltrate as an index, we found a negative association between the intracellular percentages of CD4 versus CXCL17 (R = −0.271, *P*<0.05, Fig. 3C) and a positive correlation between the intracellular percentages of CD68 and CXCL17 (R = 0.213, *P*<0.01, Fig. 3C). These results indicated that CXCL17 may negatively regulated CD4^+^ T cell infiltration while positively regulated CD68^+^ macrophage accumulation in HCC tumors.

### Prognostic Value of Combined CXCL17 Expression and Immune Cell Infiltration

Recent studies by other groups and by us have shown that immune infiltrates influence HCC progression significantly [7], [24], [29]–[31]. Immunoscore that delineate multiple immune cell infiltrate is a powerful prognostic index [32]. Therefore, we also assessed the combined influence of CXCL17 expression and immune cell densities on prognosis. Patients with more intratumoral CD4^+^, CD8^+^, CD20^+^, and CD57^+^ cells tended to have better OS and RFS, while patients with more CD15^+^ and CD68^+^ cell infiltration had less favorable prognosis (Fig. S7 and S8). In the CXCL17^high^ group, patients with more intratumoral CD4^+^, CD8^+^, CD20^+^, and CD57^+^ cells had longer survival, while those with more CD15^+^ and CD68^+^ cell infiltration had shorter survival (*P*<0.05, Fig. S7). In patients with similar immune infiltrates, for example more CD8^+^, CD20^+^, or CD57^+^ cell infiltration, CXCL17 could be used to further classify patients into subsets with different prognoses for RFS (*P* = 0.022, *P* = 0.015, *P* = 0.010, respectively, Fig. S8). For patients with higher CD20^+^ and CD68^+^ cell infiltration, CXCL17 could be used to further classify patients into subsets with different prognoses for OS, although the differences were not statistically significant (*P* = 0.064, *P* = 0.079, respectively, Fig. S7).

## Discussion

In our study, we identified CXCL17 as an independent prognostic factor for HCC after resection. Patients with less CXCL17^+^ cells within the tumor had significantly prolonged OS and RFS compared with the CXCL17^high^ subgroup. Multivariate analysis showed that tumor-infiltrating CXCL17 could also serve as a new biomarker for predicting HCC prognosis. Furthermore, analyzing the association between CXCL17 expression and immune cell infiltration revealed a significant correlation between CXCL17 expression and CD4 and CD68 infiltration. The combined CXCL17 expression and immune cell infiltration could be used to further classify patients into subsets with different prognoses for survival. These results support the idea that CXCL17 is a substantial contributor to tumor progression.

Chemokines play critical roles in recruiting a number of different cell types to the tumor microenvironment and are involved in many aspects of tumor progression [9], [33]. In the liver, the CXCL12-CXCR4, CX3CL1-CX3CR1, and CCL20-CCR6 axes have received much attention due to their regulation of tumor metastasis, tumor proliferation, and immune cell infiltration [34]. CXCL17 is a newly identified orphan chemokine whose function and clinical significance in human tumors remain unclear [17], [18]. In breast cancer and colon tumor, CXCL17 is coregulated with vascular endothelial growth factor (VEGF) and it increases vasculature *in vivo*, suggesting that it might promote tumor progression by enhancing angiogenesis [17]–[19]. CXCL17 overexpression increased the number of CD11b^+^/Gr-1^+^ myeloid-derived cells in colon tumor and was accompanied by enhanced tumorigenicity, implying that CXCL17 plays a tumor-promoting role by regulating immune cell recruitment [35]. Moreover, CXCL17 overexpression in HepG2 cells inhibits cisplatin-induced apoptosis, thus providing evidence that CXCL17 might be a potential cellular target for enhancing chemotherapy efficiency [21]. Interestingly, CXCL17 may also be involved in anti-tumor immune response. CXCL17 is upregulated in premalignant intraductal papillary mucinous neoplasm, suggesting that it acts as a danger signal during the early stages of pancreatic carcinogenesis. In malignant intraductal papillary mucinous carcinoma, CXCL17 is downregulated and is associated with decreased DC infiltration, implying that it might be one strategy for tumor escape from immune surveillance [20]. In the present study, we found abundant CXCL17^+^ cell infiltrated in HCC tumor but were relatively rare in hepatitis liver. The CXCL17 expression was a significant predictor of unfavorable RFS and OS in HCC patients, implying that CXCL17 functions as a pro-tumor factor in HCC. However, we found no correlation between CXCL17 expression and Ki-67 (Fig. S9A), a marker indicating tumor cell proliferation activity or between CD34^+^ vessel area (Fig. S9B), which indicated the angiogenesis of tumor. These data suggested that CXCL17 may not promote tumor growth or angiogenesis directly, but rather may exhibit pro-tumor abilities by regulating immunological responses in HCC.

To answer the question of whether CXCL17 regulates other immune cell subsets in HCC tumors, we evaluated the association between CXCL17 expression and local immune cell infiltration. We found a positive correlation between the intratumoral percentages of CD68 and CXCL17. CXCL17 was a chemoattractant of monocytes and macrophages. Other groups and ours have demonstrated that tumor-associated macrophage (TAM) number is a poor prognostic marker in HCC [30], [31], [36]–[38]. TAM accumulating at tumor sites exhibit immunosuppressive activity by expressing high levels of interleukin 10 and programmed death ligand 1, and promote angiogenesis by producing metalloelastase and VEGF [6], [39]. A previous study showed that CXCL17 significantly suppressed the production of proinflammatory cytokines and factors by murine J774 macrophage or primary macrophage [40]. In addition, CXCL17 induced the production of proangiogenic factors such as VEGF by treated monocytes, suggesting an indirect proangiogenic effect of CXCL17 [17]. Thus, the positive correlation between CXCL17 and macrophages in HCC indicates that CXCL17 may promote HCC progression by regulating TAM homing, cytokine production, and proangiogenic activity.

We found a negative association between the intracellular percentages of CD4 and CXCL17. However, we did not examine specific CD4 T cell subpopulations, such as T helper 1 cells, T helper 17 cells, and regulatory T cells. CD4 T cells in the tumor comprised of cell populations with both positive and negative effects on tumor immunity [29], [41], [42]. Overall, CD4 cells tended to predict favorable prognosis in HCC. Therefore, the negative association between CXCL17 and CD4 cells also indicated the pro-tumor effect of CXCL17 in HCC.

The lineage of CXCL17-producing cells in HCC has not been explored before. CXCL17 is expressed by CD68^+^ macrophages and CD138^+^ plasma cells in mucosal tissue of the intestine or bronchus [18]. By using multiple immunofluorescence staining we revealed that CXCL17 was mainly expressed by stromal cells in the peritumoral region, while tumor cells expressed CXCL17 with lower intensity. In the peritumoral stroma, neutrophils were the major source of CXCL17 production, which has not been described previously. Cells with macrophage morphology also produced CXCL17, as previously reported. To determine whether CXCL17 was induced in HCC, we also examined whether neutrophils from human peripheral blood could express CXCL17. Considering <1% of peripheral neutrophils produce CXCL17 (data not shown), CXCL17 might be induced in HCC *in situ*. To determine whether CXCL17 could be induced in HCC, we examined leukocytes from human peripheral blood exposed to 20% TSNs from two human hepatoma cells (HepG2 and Huh7). The expression of CXCL17 on neutrophils was up-regulated by TSN treatment and to a less extend by LPS stimulation (Fig. S10). Alternatively, CXCL17^+^ cells other than CXCL17^−^ neutrophils may be specifically recruited to the tumor microenvironment. CXCL17 is predicted to be structurally and functionally related to CXCL8, which is a strong, well-known chemokine for neutrophil recruitment [18]. CXCL17 expression by tumor cells was reported to recruit CD11b^+^Gr1^high^F4/80^−^ neutrophilic myeloid-derived suppressor cells [35]. Therefore, CXCL17 expression in tumor-infiltrating neutrophils implies that CXCL17^+^ neutrophils might regulate neutrophil infiltration in an autocrine manner. In addition, other groups and ours have revealed that tumor-infiltrating neutrophils are a poor prognostic factor for HCC following resection [7], [31]. Thus, CXCL17 might be responsible for neutrophil accumulation in tumor and contribute to the immunosuppressive microenvironment in the tumor.

In conclusion, we demonstrate for the first time that CXCL17 is mainly produced by tumor-infiltrating neutrophils and that it can be used as an independent indicator of poor prognosis for both OS and RFS in HCC. CXCL17 expression correlates with unfavorable immune infiltration, and combined CXCL17 and immune cell density further classifies HCC patients into subsets with different prognosis. Therefore, patients with increased CXCL17 may require closer follow-up after surgery. Further studies that define the potential regulatory role of CXCL17 in tumor-infiltrating neutrophils/macrophages in HCC may provide new insights into therapeutic strategies aimed at forming an effective immune response in HCC.

## Supporting Information

Figure S1 **Details of automated quantification method for IHC staining.** (A) Raw image acquired using the 20× objective lens on the Vectra scanner. (B) DAB and (C) hematoxylin staining unmixed according to their respective spectra. (D) Composite of unmixed stains reassigned with different colors for easier interpretation. (E) Representative image trained to establish the tissue segmentation algorithm. User-drawn training regions indicate tumor tissue (yellow), peritumoral stroma (blue), and blank (red) categories. (F) Representative compartment map following automated segmentation. (G) Object segmentation map for DAB staining (brown). The background color was removed for better visualization of the illustrated compartment. (H) Final composite map of the automated tissue and object segmentation.
(TIF)

Figure S2 **Representative images for isotype control and CXCL17 staining.** A mouse IgG2B isotype control was used for CXCL17 antibody. Bar: 20 µm.
(TIF)

Figure S3 **Representative images for double-color immunofluorescence.** Multiple staining of (A) macrophage marker CD163 (green), or (B) the T cell marker CD3 (green) CXCL17 (red), and DAPI (blue, nuclei) in paraffin-embedded sections analyzed by confocal microscopy.
(TIF)

Figure S4 **The dynamic change of CXCL17 expression in different regions of HCC tissue.** Paraffin-embedded HCC sections were stained with CXCL17 antibody. The density of CXCL17^+^ cells in nontumor (N), peritumor (P) and intratumor (T) regions of the same block were calculated (n = 227). Results are expressed as mean ± SEM (bars) of groups.
(TIF)

Figure S5 **Patients were stratified according to pathological characteristics and peritumoral CXCL17 density.** OS and RFS in relation to tumor multiplicity, tumor differentiation grade, tumor size, TNM stage, and AST and AFP levels in each subgroup were analyzed.
(TIF)

Figure S6 **Immunohistochemisty staining for the various immune cell subsets in CXCL17 low versus CXCL17 high tumors.** Immunohistochemisty for CD4 (CD4 T cells), CD8 (CD8 T cells), CD20 (B cells), CD57 (natural killer cells), CD15 (neutrophils), and CD68 (macrophages) was performed. Bar: 20 µm.
(TIF)

Figure S7 **Combined CXCL17 expression and immune cell infiltration correlated with OS.** Kaplan-Meier curves illustrating the duration of OS and RFS according to CXCL17 expression and density of CD4, CD8, CD20, CD57, CD15, and CD68 cells in the tumor region. Statistically significant differences are indicated in red.
(TIF)

Figure S8 **Combined CXCL17 expression and immune cell infiltration correlated with RFS.** Kaplan-Meier curves illustrating the duration of OS and RFS according to CXCL17 expression and density of CD4, CD8, CD20, CD57, CD15, and CD68 cells in the tumor region. Statistically significant differences are indicated in red.
(TIF)

Figure S9 **CXCL17 expression and tumor cell proliferation and angiogenesis.** Correlation between intratumoral CXCL17 expression and tumor cell proliferation rate determined by Ki-67 immunostaining (A) and tumor angiogenesis determined by CD34-vessel area (object density) (B).
(TIF)

Figure S10 **Hepatoma TSN induced CXCL17 expression in neutrophil in vitro.** Leukocytes were isolated from healthy donor peripheral blood. Cells were cultured in complete medium with 20% of tumor supernatant from HepG2 and Huh7 cells, or 50 ng/mL LPS for 12 h respectively. Control group was left untreated for 12 h. Cells were then cytospin and immunocytochemistry for CXCL17 was performed. Bar: 20 µm.
(TIF)
