# Supplementary figures and images for: CXCL17 Expression Predicts Poor Prognosis and Correlates with Adverse Immune Infiltration in Hepatocellular Carcinoma
Source: PLoS One. 2014 Oct 10;9(10):e110064. doi: 10.1371/journal.pone.0110064 (PMC4193880; doi:10.1371/journal.pone.0110064)

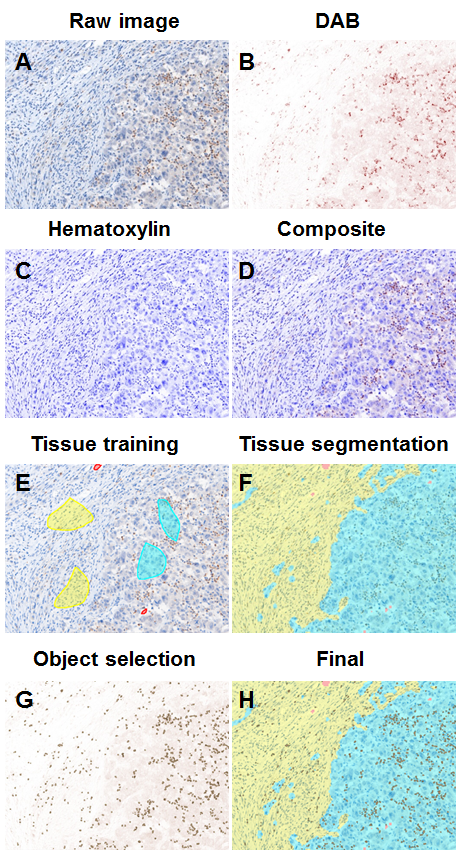

Supplement: Figure S1 — Details of automated quantification method for IHC staining. (A) Raw image acquired using the 20× objective lens on the Vectra scanner. (B) DAB and (C) hematoxylin staining unmixed according to their respective spectra. (D) Composite of unmixed stains reassigned with different colors for easier interpretation. (E) Representative image trained to establish the tissue segmentation algorithm. User-drawn training regions indicate tumor tissue (yellow), peritumoral stroma (blue), and blank (red) categories. (F) Representative compartment map following automated segmentation. (G) Object segmentation map for DAB staining (brown). The background color was removed for better visualization of the illustrated compartment. (H) Final composite map of the automated tissue and object segmentation. (TIF) [file pone.0110064.s001.tif]

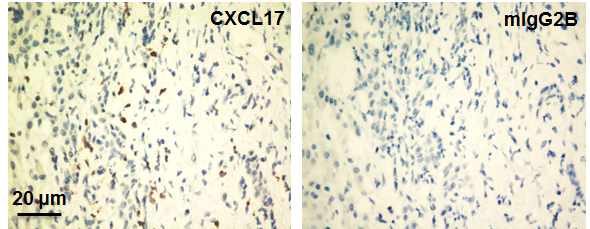

Supplement: Figure S2 — Representative images for isotype control and CXCL17 staining. A mouse IgG2B isotype control was used for CXCL17 antibody. Bar: 20 µm. (TIF) [file pone.0110064.s002.tif]

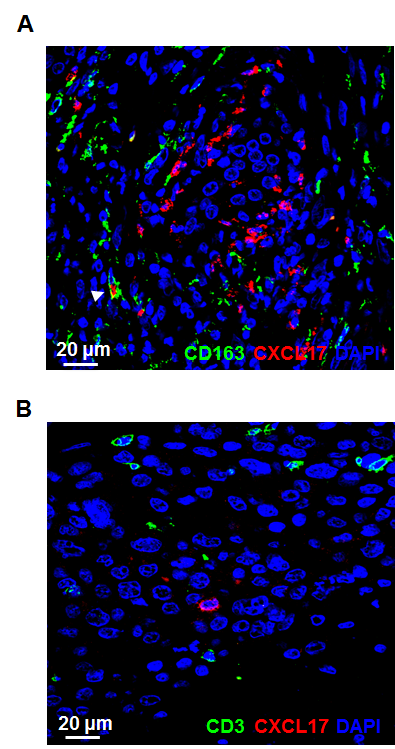

Supplement: Figure S3 — Representative images for double-color immunofluorescence. Multiple staining of (A) macrophage marker CD163 (green), or (B) the T cell marker CD3 (green) CXCL17 (red), and DAPI (blue, nuclei) in paraffin-embedded sections analyzed by confocal microscopy. (TIF) [file pone.0110064.s003.tif]

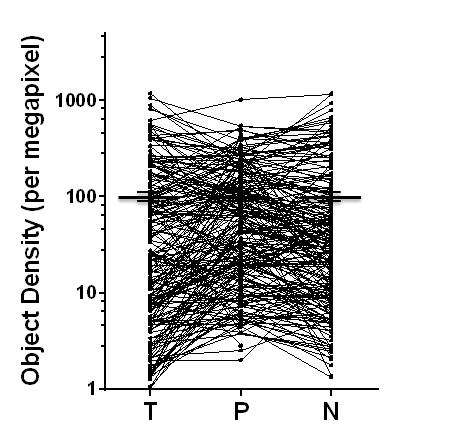

Supplement: Figure S4 — The dynamic change of CXCL17 expression in different regions of HCC tissue. Paraffin-embedded HCC sections were stained with CXCL17 antibody. The density of CXCL17+ cells in nontumor (N), peritumor (P) and intratumor (T) regions of the same block were calculated (n = 227). Results are expressed as mean ± SEM (bars) of groups. (TIF) [file pone.0110064.s004.tif]

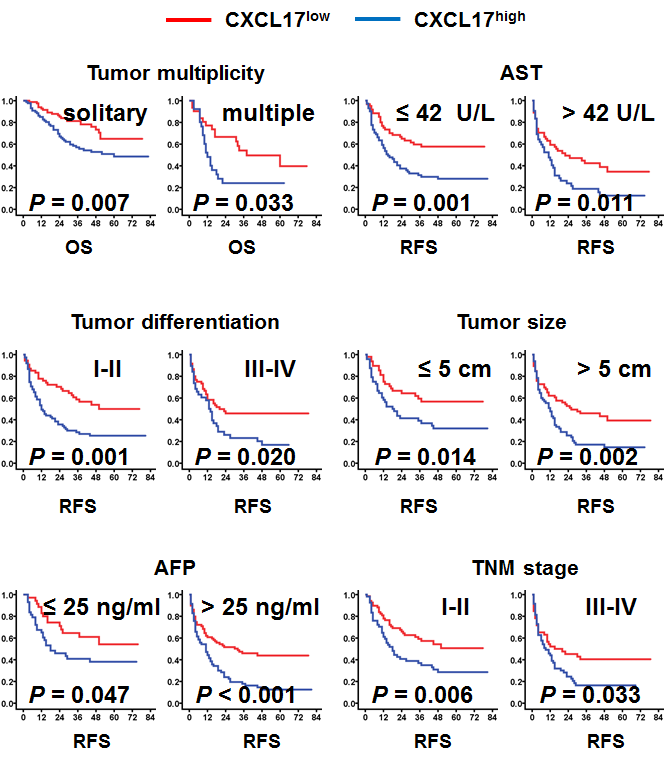

Supplement: Figure S5 — Patients were stratified according to pathological characteristics and peritumoral CXCL17 density. OS and RFS in relation to tumor multiplicity, tumor differentiation grade, tumor size, TNM stage, and AST and AFP levels in each subgroup were analyzed. (TIF) [file pone.0110064.s005.tif]

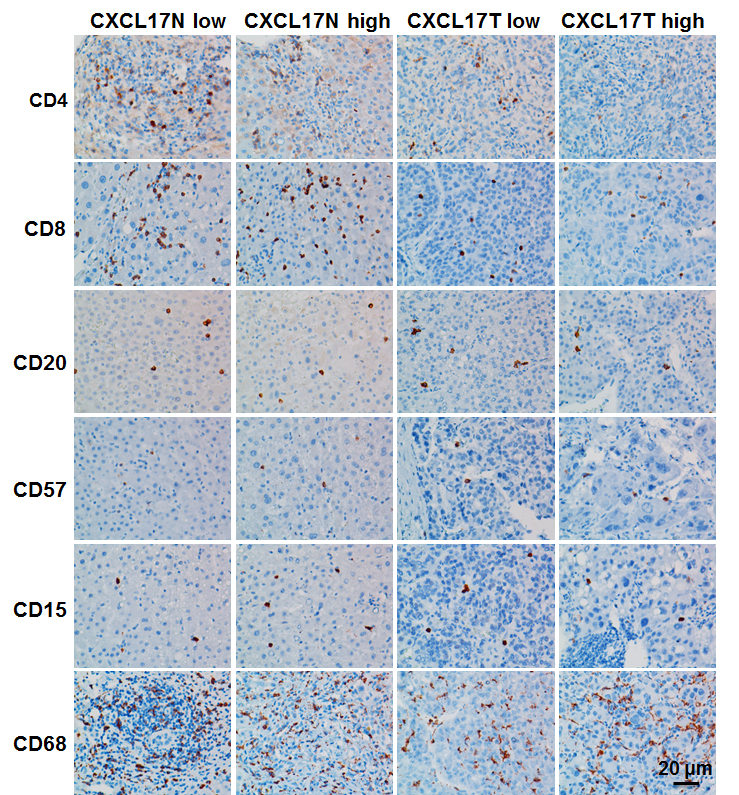

Supplement: Figure S6 — Immunohistochemisty staining for the various immune cell subsets in CXCL17 low versus CXCL17 high tumors. Immunohistochemisty for CD4 (CD4 T cells), CD8 (CD8 T cells), CD20 (B cells), CD57 (natural killer cells), CD15 (neutrophils), and CD68 (macrophages) was performed. Bar: 20 µm. (TIF) [file pone.0110064.s006.tif]

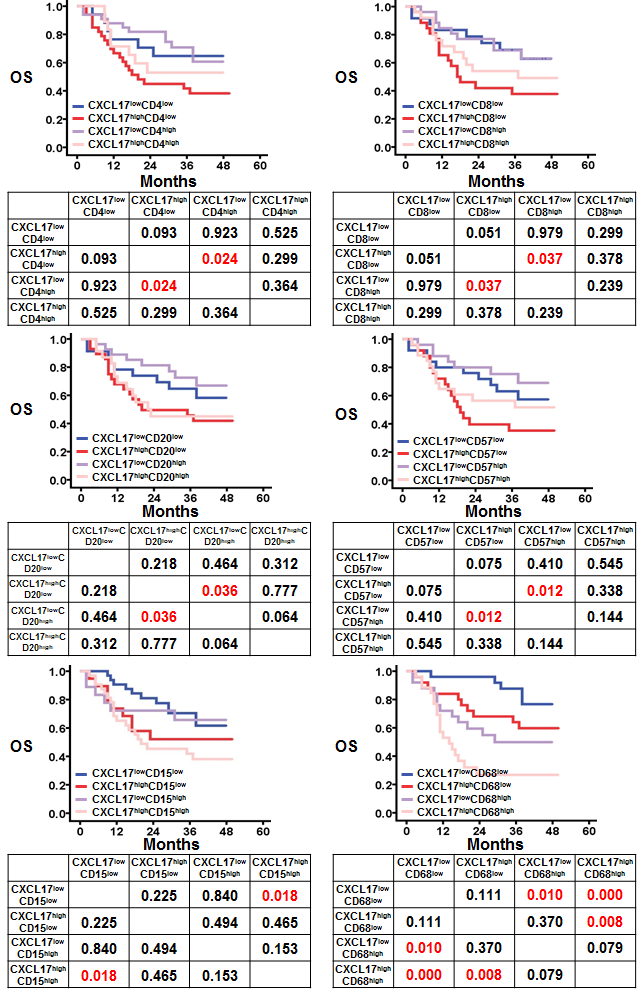

Supplement: Figure S7 — Combined CXCL17 expression and immune cell infiltration correlated with OS. Kaplan-Meier curves illustrating the duration of OS and RFS according to CXCL17 expression and density of CD4, CD8, CD20, CD57, CD15, and CD68 cells in the tumor region. Statistically significant differences are indicated in red. (TIF) [file pone.0110064.s007.tif]

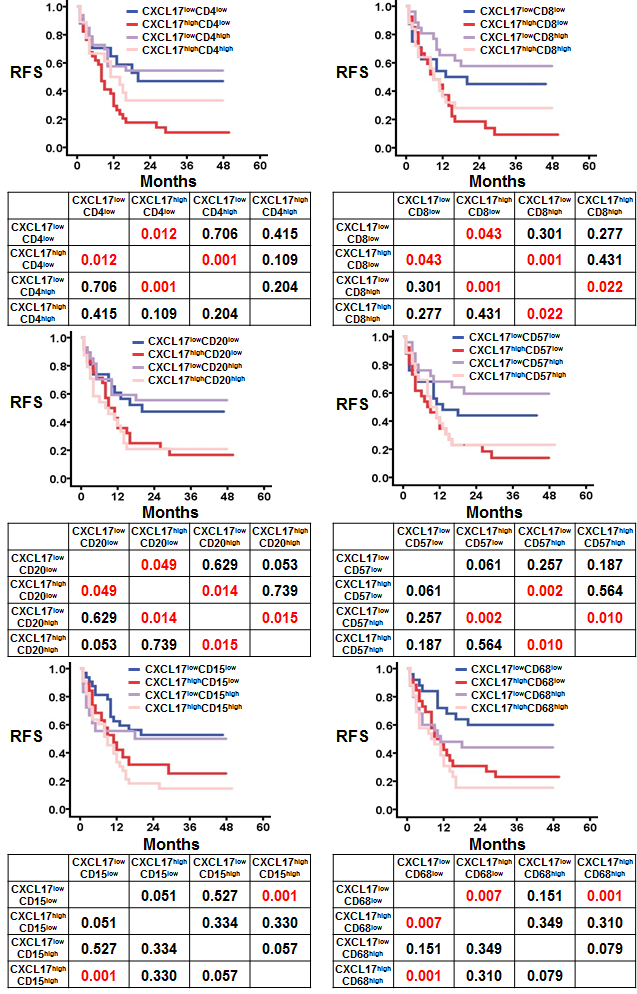

Supplement: Figure S8 — Combined CXCL17 expression and immune cell infiltration correlated with RFS. Kaplan-Meier curves illustrating the duration of OS and RFS according to CXCL17 expression and density of CD4, CD8, CD20, CD57, CD15, and CD68 cells in the tumor region. Statistically significant differences are indicated in red. (TIF) [file pone.0110064.s008.tif]

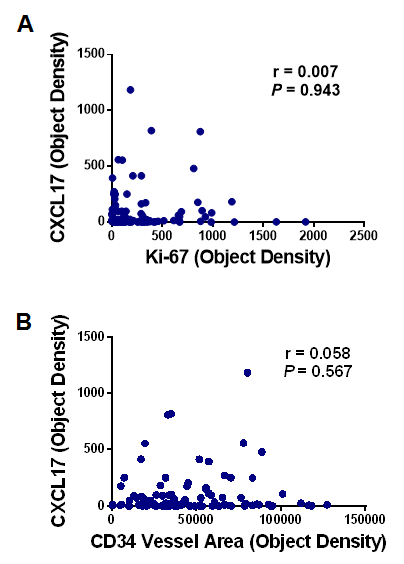

Supplement: Figure S9 — CXCL17 expression and tumor cell proliferation and angiogenesis. Correlation between intratumoral CXCL17 expression and tumor cell proliferation rate determined by Ki-67 immunostaining (A) and tumor angiogenesis determined by CD34-vessel area (object density) (B). (TIF) [file pone.0110064.s009.tif]

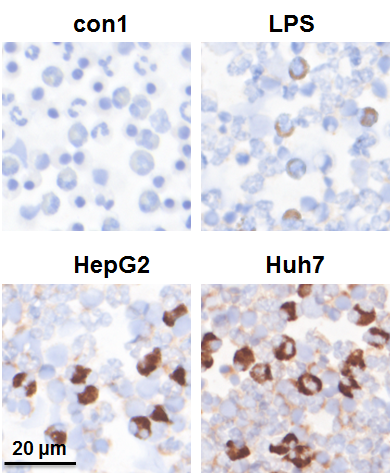

Supplement: Figure S10 — Hepatoma TSN induced CXCL17 expression in neutrophil in vitro. Leukocytes were isolated from healthy donor peripheral blood. Cells were cultured in complete medium with 20% of tumor supernatant from HepG2 and Huh7 cells, or 50 ng/mL LPS for 12 h respectively. Control group was left untreated for 12 h. Cells were then cytospin and immunocytochemistry for CXCL17 was performed. Bar: 20 µm. (TIF) [file pone.0110064.s010.tif]
